# Foreign Correspondence

**Published:** 1840-09-05

**Authors:** 


### MEDICAL EXAMINER.

DEVOTED TO MEDICINE, SURGERY, AND THE COLLATERAL SCIENCES.

No. 36.] PHILADELPHIA, SATURDAY, SEPTEMBER 5, 1840. [Vol. III.

### FOREIGN CORRESPONDENCE.

Paris, 31*st July*, 1840.

To the Editors of the Medical Examiner.

### M. ANDRAL'S RECENT EXPERIMENTS ON THE BLOOD.

The application of chemistry to medicine has received so much opposition, as well from the enlightened as the ignorant, that it becomes almost an era in science, when one in the highest rank of the profession as a practitioner, and a distinguished member of the faculty of Paris, boldly avows his conviction of its importance, and publishes valuable researches achieved under its auspices. The subject receives a new attention and fresh impulse from the reputation of the man.

At the meeting of the Academy of Sciences, held on the 26th inst., M. Andral read the result of some observations on the blood, recently made by himself, in conjunction with M. Gavaret.

It will not be impertinent, perhaps, before mentioning these, to present a short resumé of the actual state of our knowledge on this interesting point.

We all know that the principal components of the blood are albumen, fibrine, the red globules, a great number of salts, and water, and that these elementary particles are in a state very different from each other. Thus the fact is now established, that the albumen and fibrine are in solution, and that so long as the blood is healthy, they never occur in the solid form. The red globules, on the contrary, are little particles, which swim in the serum. It was for a long time believed that the fibrine, which becomes compact and forms the clot, when the sanguine fluid is beyond the influence of life, existed always in reality as a solid—that it was in a minute, subdivided state, in other words, that it was an element of the red globules. This was the opinion of MM. Prevost and Dumas, namely, that the globules and the fibrine were constituted the one by the other, and that the clot was formed by the reunion of all the globules of the blood at the moment coagulation took place without the vessels. It remained for the able physiologist of Berlin, Prof. Müller, to satisfactorily demonstrate, that the fibrine, as well as the albumen, is in a state of solution, that it is independent of the globules, and that it does not contribute to them—in fine, that the globules and the fibrine are two separate and distinct components of the blood. This fact is now established by convincing proofs, and generally admitted by biologists. Thus, after removing all the fibrine from the blood, you find all the red globules intact. Again, on retaining the globules on a filter, the fibrine coagulates in the passed liquid, entirely deprived of the globules. The fact now remains of the coagulation of the blood, due to the solidification of the fibrine, when beyond the influence of the vessels. We all know, that blood drawn from a vein, when received into a basin, soon separates into two parts—the clot and serum. This fact is, in the actual state of the science, beyond solution. It is due neither to cold, nor to repose, for, by continuing both the heat and natural movement of the blood, by artificial means, it becomes not less firmly coagulated, and in the same space of time. All that can be said is, that it is a sort of death of the sanguine fluid when beyond the vital action. The clot is always red, though the fibrine is naturally nearly colourless, but this circumstance is due to the presence of the colouring matter of the blood, which the fibrine imprisons in its meshes, in the act of coagulating. The red globules then form part of the clot, but only incidental, or as accessories, as water in a moistened sponge. The blood may consequently be described as a liquid, the components of which are albumen, fibrine, and salts, in a state of solution, and holding in suspension the little solid red globules.

The fibrine is an essential element of the blood, no matter in how small a quantity it may exist. The mean quantity in the natural state, is 5-1000, and to it appear to be due the most important properties of the blood. In fact, it results from the experiments of M. Magendie, that the blood of one animal may be injected nearly with impunity into the veins of another, provided that it be deprived of its fibrine. Hence the inconveniences arising from transfusion is not caused by the disparity in the size and form of the globules, as was once thought, but in reality from the different nature of the fibrine.

The relation of quantity between these different elements of the blood, is known to undergo variations in various diseases. What are these variations? Do we find more or less of albumen, or more or less of fibrine, or of the red globules? When and where are these augmented or decreased—in what morbid states, and under what influences, and in what bounds? The solution of these points is the object of the researches of MM. Andral and Gavaret. A number of similar analyses had been already made by Lecanu, and his results were in many respects analogous to those we are about to present our readers with, but Lecanu's experiments were conducted on the old theory—that the fibrine was a component of the globules; besides the present experiments are more numerous, more carefully executed, and the results more clear and exact.

The results are drawn from the examination of the blood of two hundred patients, and three hundred and sixty bleedings. The method was the same as that indicated by Prevost and Dumas. The variations which they have seen in the various diseases, in 1000 parts of blood, were as follows:—

| | | |
|---|---|---|
| Fibrine in the proportion of | | 1 *a* 10 |
| The red globules | " | 185 *a* 21 |
| Albumen, &c. | " | 104 *a* 57 |
| The water | " | 915 *a* 725 |

They found a simultaneous increase or diminution of the different principles in the morbid state very rare. The alterations occur singly, for the most part, but occasionally it happens that two of the elements become modified at the same time, and then it is in inverse proportion; thus, for example, if the fibrine is augmented, the red globules are diminished, and reciprocally, from which naturally results an immense change in the relation of quantity which these principles should preserve towards each other. M. Andral establishes four classes of diseases in which the natural proportions of the elements of the blood may become modified.

1. A constant augmentation of the fibrine, which may reach double the natural quantity. This occurs in the phlegmasiæ—as rheumatism, pneumonia, &c. &c.,—and even in a certain stage of phthisis pulmonalis.

2. Cases in which there is never an increase in the amount of the fibrine, but ordinarily a decrease, and what is more constant and worthy of attention, with an augmentation of the red globules. The bleedings have, for result, promptly to diminish this exaggerated proportion of them.

3. Constant diminution in the red globules. This occurs invariably in the chloroses. The decrease in the natural number of the red globules is surprising, it often descending to a quarter of the original quantity; and, what is still more remarkable, is, that the temperature of the body is preserved intact, in spite of the theory of Prevost and Dumas, upon the relation existing between the red globules and animal heat. It appears that the number of red particles has been seen notably to increase during the administration of the preparations of iron in the chloroses.

4. An appreciable diminution of the albumen in the serum. This occurs in diseases where there is an essential alteration in the blood, as the disease of Bright, and where albumen is detected in the urine. MM. Andral and Gavaret might also have added, that the albumen appears also to diminish in proportion as one bleeds in all diseases, for the specific gravity of the blood is notably diminished after each bleeding, and the density of the sanguine fluid depends neither on the fibrine or the red globules.

It often happens that several diseases co-exist, and that each one exerts special influence on the blood. This complication can always be clearly discovered in the blood. Thus, for example, a chlorotic female is attacked with pneumonia, the red globules of the blood continue small in quantity, but immediately the fibrine is augmented. "We have seen," say the authors of the memoir, "these results so constantly produced, that when we have found in the blood of a patient more than five parts of fibrine, we have never hesitated to pronounce, as existing, a complication of one of the morbid states comprised under our first division. And, on the other hand, when we have found less than two parts of fibrine, in place of five, we have not hesitated to deny this description of complication.

Independently of the disease, loss of blood and impoverished diet modify powerfully the composition of the circulating fluid, and influence the progress, duration, and type of the disorder. This fact is generally admitted by intelligent and observing practitioners, though but too frequently and fatally overlooked by others. The laws which regulate this modification are not yet established; the following are the results of the researches of our authors:

Sanguine evacuations and diet act principally on the red globules, whose quantity they diminish. In whatever diseases we practice repeated bleedings, the constant result is to render the number of red globules less and less considerable. This diminution, though constant, is not regular in its proportions in all patients. The power of resistance varies in different persons; and the loss in some persons may be in the proportion of 1 to 2, and others from 30 to 40.

Whilst the red globules are diminished by repeated bleedings, the fibrine generally preserves its natural proportion, diminishes very rarely, and, under certain circumstances, augments. Thus, when the disease is such that an increase in the quantitity of fibrine is one of its essential elements, this increase recurs, in spite of the bleedings, and in spite of the diminution of the red globules. In order that the bleedings should diminish, sensibly, the quantity of the fibrine, they must be very numerous, and the globules very considerably diminished; a condition then occurs when all the solid portions of the blood become simultaneously diminished.

After this general exposition, M. Andral proceeded to the cause of the relative alterations which occur in the first of the classes which he establishes.

This first class, as was stated before, includes those diseases where the quantity of fibrine is increased, and this augmentation has been verified in two orders of diseases—the phlegmasiæ, and in phthisis pulmonalis.

The phlegmasiæ, in which the blood has been examined, are—articular rheumatism, pneumonia, bronchitis, pleurisy, peritonitis, tonsillitis, erysipelas, cystitis, acute suppuration of the lymphatic ganglions, and in a furuncular eruption, accompanied with fever. The blood of eighty-four patients, suffering from these diseases, and of one hundred and fifty-three bleedings, was examined. In every case where the diseases existed in the acute form, and were accompanied by fever, an appreciable increase of the fibrine was always found in the blood, although subject to some variation, both in different individuals in the same disease, and also in the different diseases. Thus, in taking the number 3 as the natural mean of the fibrine, the following different degrees of elevation were found.

In acute articular rheumatism, the mean quantity of the fibrine varied between 7 and 8; the maximum was 10.

In pneumonia it was the same, offering the same minimum and maximum.

In acute bronchitis, the mean quantity was the same as in the two preceding diseases, but the maximum remained below 9.

In acute pleurisy the mean quantity of the fibrine descends, varying between 5 and 6, which last figure is its maximum, which it never exceeds, so that the lowest number in pneumonia becomes the highest in pleurisy.

In acute peritonitis the mean quantity of fibrine is the same as in pleurisy, (between 5 and 6,) the maximum 7.

In the other diseases which were the object of investigation, as acute tonsillitis, erysipelas, acute suppuration of the lymphatic ganglions, though the quantity of the fibrine was always increased, yet the mean of its numerical exponent was always a little lower than that of the preceding affections. This mean was hardly more than 5. There were, however, some examples where the maximum reached 6, and even 7.

But in no instance did the fibrine descend below 4, and very rarely below 5. Thus, in all diseases called phlegmasiæ, in which the blood has been examined, whatever was their seat, or whatever their degree of intensity, the fibrine had notably exceeded its normal number, and the limits of the scale may be indicated on one side by the figure 5, and on the other by the figure 10.

But, in order that this rule should hold good, it is necessary that both the acute stage and fever should supervene; for, if the malady is primitive, or has become so, if fever has never existed, or has disappeared, then the fibrine ceases to be in excess in the blood. As to the acute stage, the elevation of the number of the fibrine is regulated by the intensity of the local symptoms, and by that of the febrile movements.

No phlegmasia produces more fibrine than pneumonia, and, after that, acute articular rheumatism.

When the phlegmasia grows better, the fibrine diminishes. Should a relapse supervene, the increase in the fibrine reappears. If, in fine, an acute phlegmasia occurs in the course of any disease, its presence is immediately indicated by an augmentation of the quantity of the fibrine of the blood.

The fact, that the proportion of fibrine does not diminish, in spite of the number of bleedings, so long as the inflammatory condition persists—this principle appearing to be regenerated, even as one takes it away, in diminishing the mass of the sanguine fluid—is extremely curious, and we are indebted entirely for it to the observations of MM. Andral and Gavaret.

It is to be regretted, at the same time, that the attention of these observers was not directed to the modification which the fibrine undergoes in its nature, in proportion as the circulating mass is diminished. They would, we think, often not only have found it of inferior consistence, but to be deprived, in a measure, of some of its vital qualities—that is to say, that it will resist coagulation for a shorter time, after its quitting the vessels. Thus, impoverished blood—that is, the blood of the fourth and fifth bleedings—will coagulate in twelve minutes or less, instead of fifteen or eighteen minutes.

The phlegmasial state would appear to exercise no influence on the red globules; if there is any change in their condition, it is a slight diminution at the commencement. In proportion, however, as the disease is prolonged, the globules steadily decrease; this, however, is owing to the effect of the sanguine evacuations and to the diet that the patient is subjected to.

A great diminution in the number of the globules does not prevent the occurrence of an inflammation, nor from increasing, and reaching its full development. On the other hand, a great elevation in the number of the globules does not seem particularly to favour the inflammatory state. MM. Andral and Gavaret show, in fact, inflammation to be compatible with a very variable condition of the globules, from 48 to 60 even.

In the phlegmasiæ, no great alteration was observed in the other components of the blood. The water varied from 771 to 840.

We have already observed that the increase of fibrine in the blood was not confined to the phlegmasiæ, but that it was also manifested in certain stages of phthisis pulmonalis. On this point our authors have made the following observations.

Whatever be the stage of phthisis, one finds, on examination of the blood, a tendency to an unnatural increase in the quantity of fibrine, and, at the same time, a diminution in the globules. But the elevation of the first of these elements, and the second, are not equally well marked in every phase of the disorder.

So long as the tubercles are crude, the increase of fibrine is very small—the mean being about 4. The diminution of the globules at the same time, although manifest, is yet slight.

When, however, the tubercles commence to to soften, the fibrine presents a higher number, whose mean is 4½, the globules at the same time descending. In fine, when the lung is filled with caverns, the fibrine continues to increase, giving for a mean number 5, and sometimes even rising to 6, but never, however, reaching the mean number in pneumonia. When, however, the disease reaches its last stage, the fibrine commences to obey the same law of decrease which is manifest in all the other elements of the blood, and it descends below the normal standard. In general, we may say, that the greatest excess of fibrine is found in the blood of phthisical patients, at the time when a continued febrile movement is established.

Going totally in inverse ratio with the fibrine, the red globules become less and less abundant. During the earlier stages of the disease they are generally about 100, which is below their normal mean. In the second period we find them below 100; and finally, in the third stage, their quantity, in the majority of cases, becomes yet less considerable, but not descending, however, below 81. This is a very appreciable diminution, yet by no means so marked as that which takes place in chlorosis.

The other materials of the serum, in the disorder, vary from 64 to 98.

The water is most abundant in an advanced stage of the disorder. It varied from 784 to 845.

M. Andral announces the conclusion of the memoir at the next meeting.

M. Magendie then rose, and announced to the Academy that he had for some years back been employed upon similar investigations, and that he would now place in the custody of the society, the tables, the results of these observations, and from which he proposed, at a later period, to draw his conclusions.

